# Butylboronic acid promotes cell proliferation, alkaline phosphatase activity, and expression of *GJA1*, *ALP*, and *NaBC1* in human dental pulp cells *in vitro*

**DOI:** 10.3389/fdmed.2025.1518389

**Published:** 2025-02-11

**Authors:** Takashi Nakano, Hidenori Hamba, Takashi Muramatsu

**Affiliations:** Department of Operative Dentistry, Cariology and Pulp Biology, Tokyo Dental College, Tokyo, Japan

**Keywords:** boron, cell differentiation, cell proliferation, dental pulp cells, resin, GJA1, ALP, NaBC1

## Abstract

The objective of this study is to investigate the effects of n-butylboronic acid (n-BA) on the proliferative activity, alkaline phosphatase activity, expression of hard tissue formation related genes, *ALP* and *GJA1*, and boron transporter gene, *NaBC1*, in human dental pulp cells. Human dental pulp cells were spread in culture medium with n-BA. The proliferative activity was increased in n-BA group. alkaline phosphatase staining and alizarin red S staining identified no obvious differences between the n-BA(+) and n-BA(−) groups. Alkaline phosphatase activity was significantly increased in the n-BA(+) group compared to the n-BA(−) group. Expressions of *GJA1*, *ALP*, and *NaBC1* in the n-BA(+) group were higher than that of the n-BA(−) group. There were no significant differences between NaBC1-shRNA-transfected group and control group about proliferation and alkaline phosphatase activity. The results suggest that n-BA promotes cell proliferation, alkaline phosphatase activity, and expression of *GJA1*, *ALP*, and *NaBC1*, in human dental pulp cells.

## Introduction

Direct pulp capping is one of the treatments to conserve dental pulp tissue by covering the exposed pulp with a biocompatible material, blocking external stimuli, and forming tertiary dentin. Calcium hydroxide has been well-used material for direct pulp capping. Currently, calcium mineral trioxide aggregate (MTA), a major calcium silicate cement, is the primary material used for this treatment (1). The materials have a high pH, antibacterial properties, and induces tertiary dentin formation (2). MTA promotes tertiary dentin formation and seals the exposed pulp area tightly due to hydraulicity (2, 3).

4-methacryloxyethyl trimellitic anhydride/methyl methacrylate-tri-n-butyl borane (4-META/MMA-TBB) resin, an adhesive resin cement, adheres firmly to dentin in moist cavities and restorations (4). Although 4-META/MMA-TBB resin is cytotoxic before polymerization, it becomes non-cytotoxic after polymerization and exhibits wound-healing properties (5). However, wound healing of the dental pulp may not be actively promoted by 4-META/MMA-TBB resin due to the lack of bioactive component. Therefore, a recent study has aimed to explore the bioactive components into 4-META/MMA-TBB resin such as hydroxyl apatite inducing hard tissue formation (6).

Oxidized tri-n-butyl borane (TBB-O), a cytotoxic compound and one of the organoboron compounds, is included in the 4-META/MMA-TBB resin as polymerization initiator. After polymerization, TBB-O is assumed to convert to stable boric acid. When TBB-O contacts with air, boron radicals are generated, and after the radicals contact with water, boronic acid is generated (7). Thus, TBB-O may have the possibility of conversion to boron, but the detailed reaction mechanism remains unknown (8). Boron plays important roles such as promoting brain function, wound healing, and bone growth in humans (9). Intracellular boron transport is assumed to be through passive diffusion, although a boron transporter, sodium borate cotransporter 1 (NaBC1), was identified in mammalian cells (10, 11). Recently, materials with low boron concentrations have attracted attention as promote differentiation and hard tissue formation of osteoblasts, and are expected to be applied as bioactive materials (12–15). Therefore, we hypothesized that the incorporation of a non-toxic organoboron compound like boric acid into the 4-META/MMA-TBB resin may promote cell proliferation activity and hard tissue formation in dental pulp cells. To understand the effect of the organoboron compound to dental pulp cells, we investigated cell proliferative activity, alkaline phosphatase (ALP) activity, hard tissue-related gene expression, and hard tissue formation in cultured human dental pulp cells incorporated with organoboron compound. We also analyzed NaBC1 expression to elucidate links between human dental pulp cell activity and organoboron.

## Materials and methods

**Figure 1:**
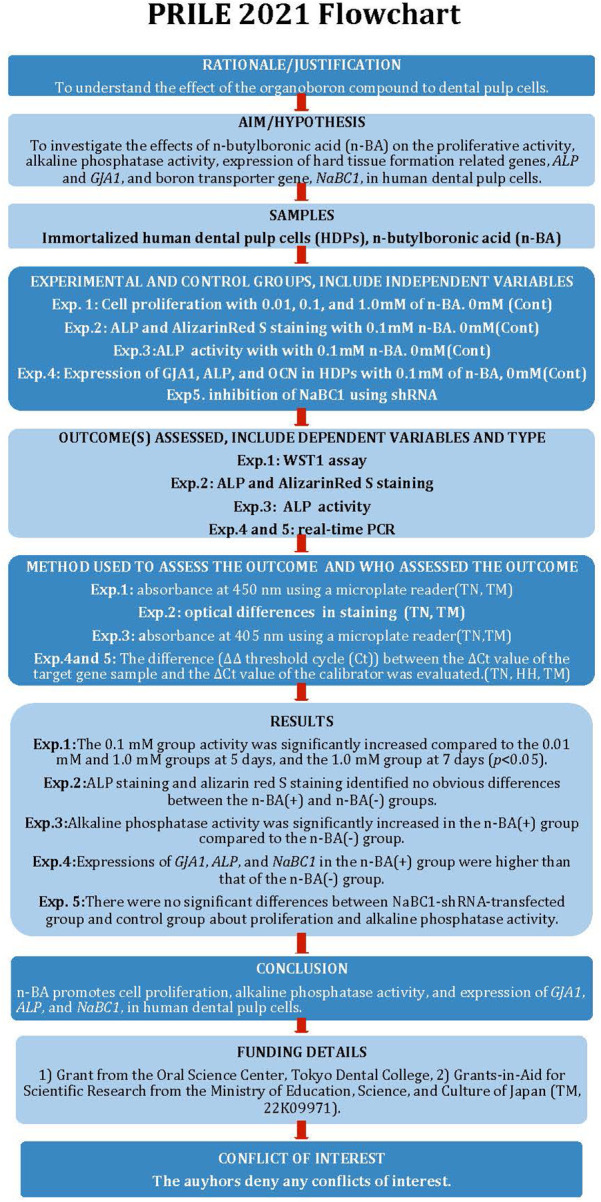
The PRILE 2021 flowchart.

The manuscript of this study has been written according to Preferred Reporting Items for Laboratory studies in Endodontology (PRILE) 2021 guidelines (16). Figure 1 is a flow chart based on the guidelines.

### Cell culture

Immortalized human dental pulp cells (HDPs) transfected with the telomerase transcriptase gene were used. These cells were provided by Prof. Takashi Takata (Hiroshima University Graduate School, Hiroshima, Japan; Shunan University, Shunan, Japan); their characteristics are previously described (17). Cells were cultured in growth medium (GM), alpha minimum essential medium (αMEM; Thermo Fisher Scientific, Waltham, USA) supplemented with 10% fetal bovine serum (FBS; Cytiva, Tokyo, Japan), and 100 IU/ml penicillin-streptomycin (Thermo Fisher Scientific) with passaging before subconfluence. Cell culture was performed under humidified conditions at 37°C, 5% CO_2_, and the culture medium was changed every 2 days.

### Preparation of organoboron compounds

We used n-butylboronic acid (n-BA; Sigma-Aldrich, St. Louis, USA), an organoboron compound chemically like boric acid. The n-BA was prepared at 0.1 g/ml by dissolving in αMEM, filtered through 0.2 µm filter (Sartorius AG, Göttingen, Germany) and stored. The n-BA was diluted to the final concentration in the culture medium selected for each experiment.

### Cell proliferation

HDPs were seeded on 96-well plates (IWAKI, Tokyo, Japan) at a density of 1.5 × 10^3^ cells/well and exchanged with GM supplemented with n-BA 24 h later. Final concentrations of 0.01, 0.1, and 1.0 mM of n-BA GM were prepared. As a control group, growth medium without n-BA was prepared. Cell proliferation activity was measured by the tetrazolium salts (WST-1) assay (Roche Diagnostics, Mannheim, Germany) according to the manufacturer's instructions. The reagent was added to wells at 3, 5, and 7 days with GM at a determined n-BA concentration, and incubated at 37°C for 60 min, and absorbance was measured at 450 nm using a microplate reader (Agilent Technologies, California, USA).

### Differentiation of HDPs

To investigate odontogenic differentiation, HDPs were seeded on 6-well plates at a density of 1.0 × 10^5^ cells/well. After incubation at 37°C, 5% CO_2_ humidified until 70%–80% confluency, the medium was changed to osteogenic differentiation medium (OBM). OBM was prepared by adding 10% FBS (Cytiva), 100 IU/ml penicillin-streptomycin (Thermo Fisher Scientific) 10 nmol/L dexamethasone (Sigma-Aldrich), 10 mmol/L β-glycerol phosphate (Wako Pure Chemical Industries, Osaka, Japan), and 50 mmol/L ascorbic acid (Wako Pure Chemical Industries) to αMEM. The culture medium was changed every 2 days.

### ALP and alizarin red S staining

To evaluate ALP production or mineralization, HDPs were cultured in OBM with and without n-BA. After 7, 14, 21, and 28 days in each culture medium, HDPs were rinsed with phosphate buffered saline (PBS; KAC, Hyogo, Japan) and fixed in 4% paraformaldehyde solution (Wako Pure Chemical Industries) for 10 min. For the ALP staining, TRAP/ALP staining kit (Wako Pure Chemical Industries) was used according to the manufacturer's instructions. HDPs were rinsed with PBS and permeabilization with ethanol/acetone (50:50 v/v) was carried out. ALP substrate solution was incubated at 37°C for 60 min, and the reaction quenched by rinsing with PBS. For staining with alizarin red S (Wako Pure Chemical Industries) after fixation with 4% formaldehyde solution for 10 min, HDPs were stained with alizarin red S staining solution (pH 6.38 with ammonia solution, Wako Pure Chemical Industries) for 60 min, and the reaction quenched by washing with Milli-Q water. The stained HDPs were observed and photographed using an all-in-one fluorescence microscope BZ-X710 (Keyence, Osaka, Japan).

### ALP activity

ALP activity changes with and without n-BA were quantified for HDPs. HDPs were cultured with and without n-BA for 7, 14, 21, and 28 days. ALP activity was evaluated using LabAssay ALP (Wako Pure Chemical Industries) according to the manufacturer's instructions with absorbance measured at 405 nm using a microplate reader. To extract total protein, HDPs were dissolved in radio-immunoprecipitation assay buffer (1% Nonidet P-40; Sigma-Aldrich, 150 mmol/L NaCl, and 50 mmol/L Tris; pH = 7.4; containing protease inhibitors). Protein concentrations were determined by the Lowry method using the DC Protein assay (Bio-Rad Laboratories, Hercules, Canada) with bovine serum albumin as standard. ALP activity was calculated as unit per μg protein.

### Real-time reverse transcription-polymerase chain reaction (real-time PCR)

The expression of hard tissue-associated and NaBC1 genes was analyzed by real-time PCR of HDPs. HDPs were cultured in OBM with an n-BA concentration of 0.1 mM [n-BA(+)] or without n-BA [n-BA(−)]. At each time point, total RNA was extracted using TRIzol reagent (Thermo Fisher Scientific) according to the manufacturer's instructions. The RNA was reverse transcribed to complementary DNA using the High-Capacity cDNA Reverse Transcription Kit (Applied Biosystems, Foster City, Canada). Analysis of RT-PCR was performed using 2X TaqMan Fast Universal PCR Master Mix no AmpErase reagent (Applied Biosystems) on an ABI7500 Fast System (Applied Biosystems). *Gap junction protein alpha 1* (*GJA1*, Hs00748445_s1), *ALP* (Hs01029144_m1), *osteocalcin* (*OCN*, Hs01587814_g1), and *NaBC1* (Hs00984700_g1) were used as target genes and glyceraldehyde-3-posphate dehydrogenase (*GAPDH*; Applied Biosystems) as an endogenous control gene. The polymerase chain reaction consisting of denaturation at 95°C for 3 s then annealing and extension at 60°C for 30 s was performed for 40 cycles. The incubation period was 7, 14, and 28 days for hard tissue-related gene analysis, and 21 and 28 days for *NaBC1* analysis. Relative expression of target genes was estimated using the ΔΔ threshold cycle (Ct) method. The Ct value of *GAPDH* was subtracted from the Ct value of the target gene to normalize the amount of messenger RNA (mRNA) of the target gene to obtain the ΔCt value. The difference (ΔΔCt) between the ΔCt value of the target gene sample and the ΔCt value of the calibrator was evaluated.

### Inhibition of NaBC1 with shRNA

To understand involvement of NaBC1, shRNA was performed. In brief, target sequence of *NaBC1* (*SLC4A11*), CCCTACATGAAGATGATCTTT, was inserted into the shRNA vector (pLKO.1-puroLentivirus vector, Sigma-Aldrich) and the vector was transfected into the HDP at multiplicities of insertion (MOI) of approximately 5. After transfection of the vector, the effect was confirmed WST-1, ALP activity, ALP staining, and alizarin red S staining. As a negative control, non-target shRNA control transduction was used (Sigma-Aldrich).

### Statistical analysis

Statistical analysis was performed in IBM SPSS 28.0 J for Windows (SPSS Japan, Tokyo, Japan).

We conducted a normality test using the Kolmogorov-Smirnov test, which indicated that the data followed a normal distribution. A Levene test was performed on the WST-1 values, which showed homogeneity of variance. After performing a one-way ANOVA, we carried out Tukey's test. For the ALP activity and real-time PCR, we used a *t*-test to compare the two groups. Significance was set at *p* < 0.05.

## Results

### Cell proliferation

**Figure 2:**
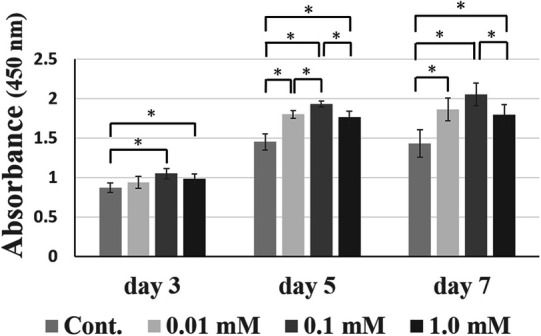
WST-1 assay in HDPs. WST-1 activity was carried out at 3, 5, and 7 days in GM with different n-BA concentrations (0, 0.01, 0.1, and 1.0 mM). WST-1 activity for the 0.1 mM n-BA concentration was the highest statistically in HDPs. Data represent means ± standard deviations (SD) (*n* = 5). Significantly different at *p < 0.05.

The HDPs proliferation activity when cultured with different n-BA concentrations was evaluated (Figure 2). For all periods, the 0.1 mM group showed significantly increased activity compared to the control group (*p* < 0.05). The 0.1 mM group activity was significantly increased compared to the 0.01 and 1.0 mM groups at 5 days, and the 1.0 mM group at 7 days (*p* < 0.05). Based on the cell proliferation assay results, we employed 0.1 mM n-BA for the present study, because of the most increase at 0.1 mM n-BA concentration in this experiment.

### ALP staining and alizarin red S staining

To evaluate the formation of hard tissue by incorporation of n-BA, ALP staining and alizarin red S staining were performed.

**Figure 3:**
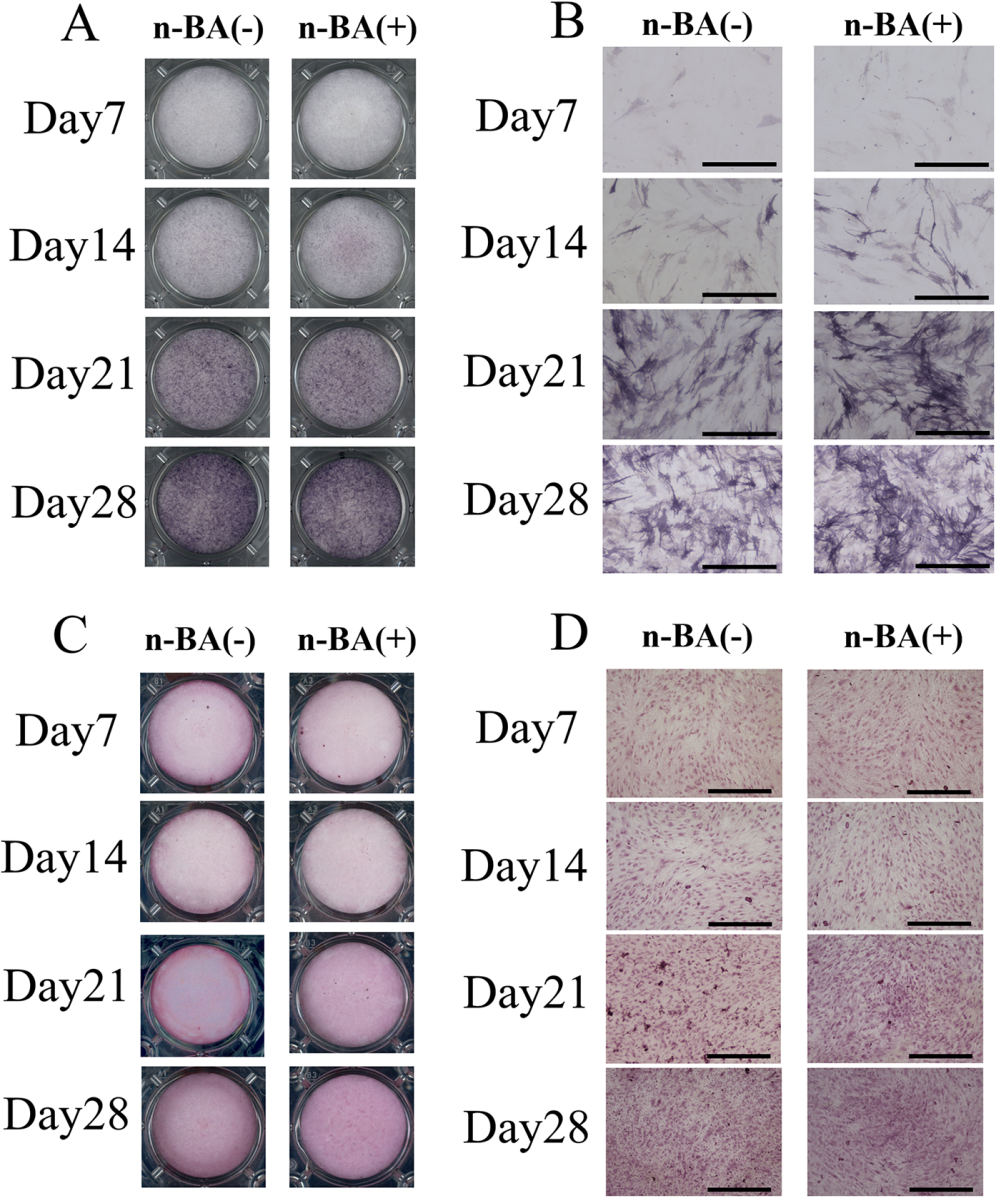
ALP and ARS staining. HDPs were cultured with 0.1 mM n-BA for 7, 14, 21, and 28 days, then either ALP staining, or ARS staining performed. In ALP staining, the n-BA(−) and n-BA(+) groups showed improved staining over time. ARS staining showed improved staining in the n-BA (+) group compared to the n-BA (−) group on days 21 and 28. **(A)** Dish images of ALP staining, **(B)** Optical microscope images of ALP staining, **(C)** Dish images of ARS staining, **(D)** Optical microscope images of ARS staining, Bar = 500 μm, (*n* = 4).

ALP staining indicated the stained area spread over time for both the n-BA(−) and n-BA(+) groups (Figure 3A). At 14, 21, and 28 days, both groups stained uniformly. Optical microscopic images showed cytoplasm staining in the spindle-shaped cells (Figure 3B). In ARS staining, the dish images exhibit no definite differences in staining between both groups and time points (Figure 3C). Optical microscopic images also showed no cell staining in both groups, but each showed an improvement in staining over time (Figure 3D).

### ALP activity

**Figure 4:**
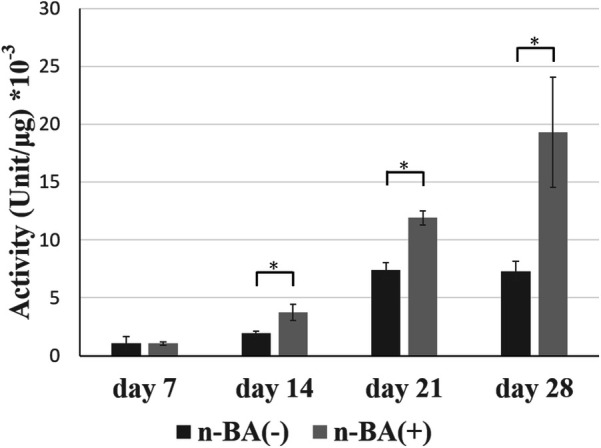
ALP activity in HDPs. ALP activity of HDPs cultured for 7, 14, 21, and 28 days in medium prepared with n-BA at a final concentration of 0.1 mM. At 14, 21, and 28 days, OBM with n-BA increased the ALP activity in HDPs. Data represent means ± standard deviations (SD) (*n* = 4). Significantly different at *p < 0.05.

ALP activity changes were evaluated for HDPs cultured with and without n-BA (Figure 4). ALP activity tended to increase over time for both the n-BA(−) and n-BA(+) groups. The ALP activity of the n-BA(+) group was significantly increased compared to the n-BA(−) group at 14, 21, and 28 days (*p* < 0.05) (Figure 4).

### Expression of *GJA1*, *ALP*, and *OCN*

Expression of hard tissue formation-related genes (*GJA1*, *ALP*, and *OCN*) in HDPs were analyzed, with mRNA expression detected in all groups.

**Figure 5:**
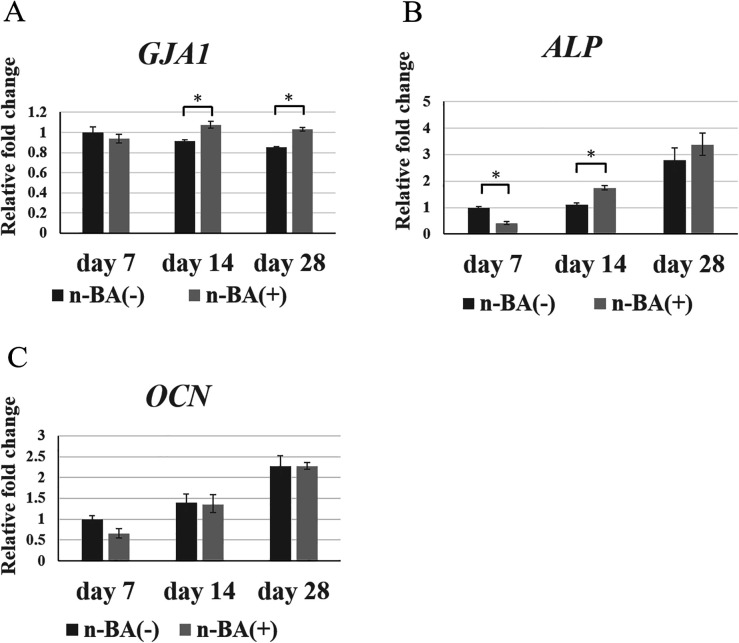
*GJA1*, *ALP*, and *OCN* expression in HDPs. The mRNA expression of *GJA1*, *ALP*, *OCN*, in each graph was normalized against that of GAPDH. The n-BA increased expressions of *GJA1* at 14 and 28 days and *ALP* at 14 days, but did not change the expression of *OCN* at all time points. Data represent means standard deviations (*n* = 4). Significantly different at *p < 0.05. **(A)** *GJA1*; **(B)** *ALP*; **(C)** *OCN*.

Expression of *GJA1* was not different between the n-BA(+) and n-BA(−) groups at 7 days, whereas the expression in the n-BA(+) group was 1.2-fold that of the n-BA(−) group and was significantly higher at 14 and 28 days (*p* < 0.05) (Figure 5A). *ALP* expression in the n-BA(+) group was significantly lower than in the n-BA(−) group at 7 days (*p* < 0.05), while at 14 days it was 1.6-fold significantly higher than in the n-BA(−) group (*p* < 0.05) (Figure 5B). Expression of *OCN* was not significantly different between the n-BA(+) and n-BA(−) groups at all time points (Figure 5C).

### Expression of *NaBC1*

Expression of *NaBC1* in HDPs were analyzed, with mRNA expression detected in all groups.

**Figure 6:**
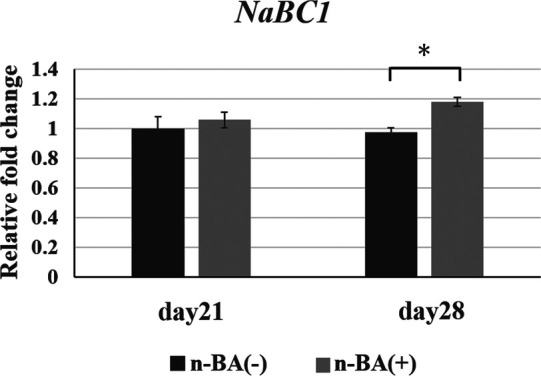
*NaBC1* expression in HDPs. The mRNA expression *NaBC1* in graph was normalized against that of *GAPDH*. The n-BA increased expressions of *NaBC1* at 28 days. Data represent means standard deviations (*n* = 4). Significantly different at *p < 0.05.

Expression of *NaBC1* in the n-BA(+) group was not different to that in the n-BA(−) group at 21 days, although the expression in the n-BA(+) group at 28 days was significantly increased 1.2-fold (*p* < 0.05) (Figure 6).

### Inhibition of NaBC1

**Figure 7:**
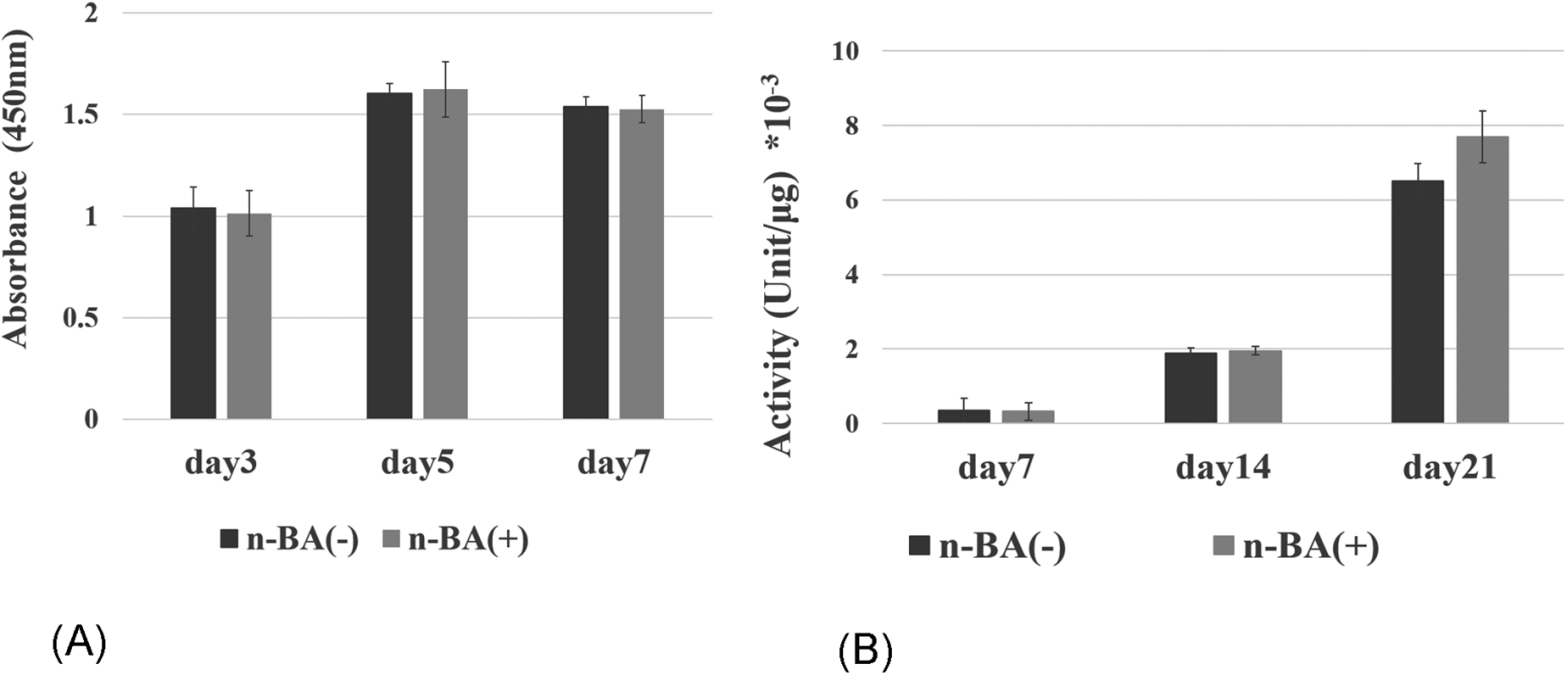
Inhibition of NaBC1 with shRNA. **(A)** Proliferative assay: There were no significant differences between NaBC1-shRNA-transfected group and control group at 3, 5 and 7 days (*n* = 4). **(B)** ALP activity assay: No significant differences were seen between NaBC1-shRNA-transfected group and control group at 7, 14 and 21 days (*n* = 4).

The proliferation of HDPs that NaBC1-shRNA was transfected with 0.1 mM n-BA was evaluated using WST-1 assy. There were no significant differences between NaBC1-shRNA-transfected group and control group at 3, 5 and 7 days (Figure 7A). ALP activity also showed no significant differences between NaBC1-shRNA-transfected group and control group at 7, 14 and 21 days (Figure 7B).

## Discussion

We investigated effects of n-BA, an organoboron compound similar to TBB-O, on cell proliferation, ALP activity, expression of hard tissue-related genes, and hard tissue formation in dental pulp cells *in vitro*. Furthermore, expression of *NaBC1*, a boron transporter, was also elucidated.

Although TBB-O is a cytotoxic compound (8), 4-META/MMA-TBB resin, after polymerization, becomes biocompatible with dental pulp tissue (5). On the other hand, the cell proliferative effects of boron are reported in human osteoblasts (13), thus the cytotoxicity of n-BA appears elusive and controversial. In this study, incorporation of 0.1 mM n-BA significantly increased HDPs proliferative activity compared to the control and that of 1.0 mM n-BA. An earlier study demonstrated increased cell viability in human dental pulp stem cells with the incorporation of 10–20 µg/ml of sodium pentaborate pentahydrate (NaB), an n-BA-like boron-containing compound, but concentrations of 50 µg/ml and above decreased cell viability (18). The present results are consistent with the earlier study, suggesting an effective concentration of n-BA is required for maximizing HDPs proliferative activity.

### ALP activity, hard tissue formation, and differentiation of HDPs

Incorporation of n-BA significantly increased ALP activity in HDPs compared to the control at 14, 21, and 28 days. ALP activity of bone marrow stem cells was significantly increased with low concentrations of boric acid, enhancing osteogenic effects (12, 19). Moreover, human dental pulp stem cells with 20 µg/ml NaB significantly increased ALP activity at 14 days (18). Our results suggest that n-BA promotes ALP production in human dental pulp cells.

We investigated the expression of hard tissue formation-related genes (*GJA1*, *ALP*, and *OCN*) with and without n-BA by real-time PCR in this study. *GJA1* encodes a gap junction protein, connexin43 (CX43), with an important role in odontoblastic differentiation (20, 21). ALP is synthesized from early differentiation during hard tissue formation, while OCN is synthesized primarily at the late stage of differentiation in osteoblasts (22). The real-time PCR results showed that n-BA increased the expression of *GJA1* and *ALP* in HDPs, but did not change *OCN* expression. We previously investigated dental pulp viability by determining ALP activity and CX43 (23–25). ALP is an enzyme expressed in early-stage mineralization, and is a dental pulp cell viability marker. Also, we previously observed higher expressions of CX43 in young odontoblasts in rats and in young dental pulp in humans, suggesting that this reflected dental pulp cell viability (20, 26). Furthermore, dental pulp cells regulate cell proliferation via gap junctions mediated by CX43 (24). Therefore, we believe that this increase in ALP activity and expression of *GJA1* and *ALP* by n-BA incorporation, in this study, indicates increased HDPs viability. On the other hand, studies using dental pulp stem cells or bone marrow stromal cells showed increased expression of *ALP* and *OCN* after 7 days with boric acid or NaB (18, 19), but these results do not correspond to the results of this study. The likely reason is that the dental pulp stem cells are mesenchymal stem cells that become differentiated mature cells for hard tissue formation, synthesizing OCN after stimulation. In general, differentiation markers such as ALP and OCN are lower during the proliferative phase. Therefore, the mRNA expression of *ALP0* and *OCN* in n-BA(+) group would downregulate at 7 days. Furthermore, cell staining revealed no significant changes in alizarin red S staining of HDPs with n-BA compared to the control group for up to 28 days. The exact reasons remain unknown, but other factors are required for hard tissue formation after n-BA incorporation in HDPs.

We investigated the expression of *NaBC1*, a boron transporter, using HDPs in the presence of n-BA. Boron promotes differentiation and hard tissue formation in human osteoblastic cells (12, 13), by activating the Wnt/β-catenin pathway (13, 27). Borax is reported to stimulate NaBC1 in mouse mesenchymal stem cells promoting osteogenesis via the BMP pathway (28). However, there are no previous reports investigating NaBC1 gene expression in dental pulp cells. In this study, real-time PCR results demonstrated higher expression of *NaBC1* in HDPs with n-BA at 28 days. We found that n-BA increased ALP activity in HDPs, and our data have shown a decline in proliferation assay and ALP activity in HDPs using *NaBC1* shRNA. Although it is not definite whether the boron in n-BA is directly involved in cell proliferation and ALP activity under the conditions of this study, the results imply correlation between NaBC1 and cell proliferation or ALP activity.

## Conclusion

**Figure 8:**
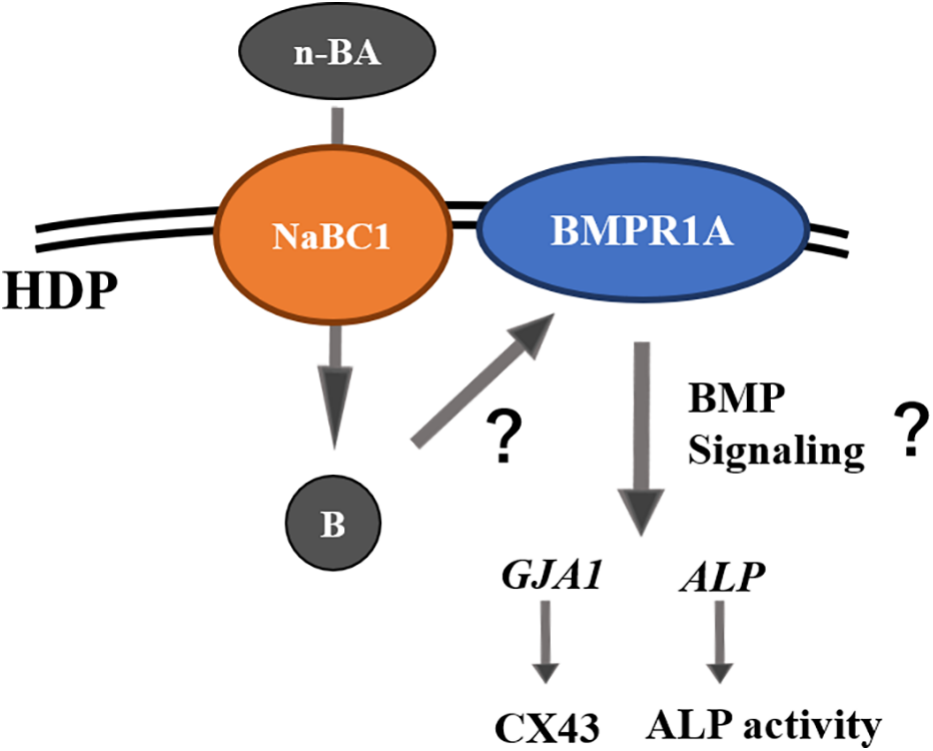
Schematic diagram of pulp cell responses under n-BA incorporation assumed by the results of this study.

Incorporation of 0.1 mM n-BA increased cell proliferation, ALP activity and gene expression of *GJA1* and *ALP* in HDPs, but hard tissue formation was not promoted. Expression of NaBC1 stimulated by n-BA incorporation would be involved in the increase of ALP in HDPs as shown in the schema (Figure 8).

## Data Availability

The original contributions presented in the study are included in the article/Supplementary Material, further inquiries can be directed to the corresponding author.
